# Our Contemporaries

**Published:** 1912-03

**Authors:** 


### OUR CONTEMPORARIES

THE KETTLE GETS BACK AT THE POT.—The *Journal of the A. M. A.* pays us a very handsome compliment for our reading matter, while protesting against our carrying the advertisement of Duffy Malt Whiskey which is said to be advertised as a consumption cure. Our readers will note that the advertisement which we carry, as well as those of the other journals criticised, so far as we have been able to consult them, are perfectly proper, conservative statements of generally accepted alcoholic therapeutics. Under ideal conditions, alcoholic beverage should be used only as drugs or adjuvants and should be advertised only in medical journals. Under existing conditions, they are not so used and we cannot refuse properly worded advertisements of reputable firms on the basis of lay advertising, any more than we can those of manufacturers of soaps, foods, or strictly commercial wares. The *British Medical Journal* has, we are informed, accepted the Duffy advertisement after investigation. The only advertisement of this firm that we have found among many newspapers and magazines, is one advising, at the beginning of a cold, to go to bed and take a hot lemonade with a small quantity of whiskey (Duffy's of course.) This is generally accepted advice and comes well within the field of domestic therapeutics.

But we have found that the *Journal of the A. M. A.* carries advertisements of medicinal soap, foods, beverages, cod liver oil, etc., which are advertised to the laity—not to go back beyond the very recent ethical advertising code of that Journal. There is no difference in ethics that we can see between advertising whiskey as a consumption cure and advertising another milder beverage as "a true tonic without an evil aftermath," or advertising cures of skin diseases, or dwelling on the nutrient wonders of prepared foods which the profession employs in feeding delicate infants, serious digestive cases, etc. As to cod liver oil, the advertisement which the *Journal of the A. M. A.* displays so conspicuously and which is essentially duplicated in a number of popular magazines, does not mention tuberculosis but it does not need to. The laity associates this disease and this food—or drug—without present instruction.

Just as a matter of interest and not as an expression of personal opinion nor as an endorsement of the policy of advising lay use of medicaments even on a basis of fact, we would recall that it is not many years since alcoholic beverages were pretty generally regarded by the profession not merely as adjuvants in diet but as distinctly curative in tuberculosis.

In the last six months, we have sacrificed about $200 worth of advertising on ethical grounds, but we cannot place nice ethical distinctions higher than common honesty in fulfilling business contracts. The *Journal of the A. M. A.* has a support which is practically unlimited, independently of advertisements. It is making a large annual profit, both from advertisements and from the dues which are considerably more than are necessary for the maintenance of the organization and its publications. An independent journal, on the other hand, is supported mainly by its advertisements. Notwithstanding this fact, we may say that our policy in regard to the BUFFALO MEDICAL JOURNAL is precisely the same as in regard to practice of medicine. If it cannot be maintained ethically and on a satisfactory plane of professional usefulness, it will not be continued. Fortunately, there is no prospect of failure; on the contrary there has been an increase of professional and business support which warrants full confidence in an indefinite term of usefulness.

Not to dodge an issue, however, we may say that a periodical is a public institution. It is impracticable to make everything in it, in every department, conform to our own or any one else's views. To do so would be contrary to the spirit and probably, the letter of the law, if anyone chose to contest the point. In making this qualification, we wish to guard explicitly against its interpretation as a disposition to violate principles of right and wrong or even of ethics in the broad sense.

---

READ the true story of the "Man in the Iron Mask" in the *Maltosia Monthly* for Jan. 1912.

---

THE *American Journal of Clinical Medicine* for Jan. begins its nineteenth year auspiciously. Dr. Thomas E. Moss of the Philippines describes an operation for vesical calculus, upon a Kalinga chief and gives an interesting description of the country, with pictures. Many other interesting articles appear.

---

THE "Bloodless Phlebotomist" contains a case report by Dr. S. A. Keene of Baltimore, in which an application of antiphlogistine removed many small shot, facilitated the subsequent extraction of others, and reduced inflammation Various other reports include favorable results in pneumonia, mastitis, tuberculous glands (by inducing hyperaemia), pleurisy, lumbago, joint effusions, etc. In this connection we may mention a case of long standng facial wen, deeply seated, as large as a pea, which disappeared after a few applications of antiphlogistine and has not reappeared in five years.

---

THE *Medical Times* of New York, so long under the able management of Dr. A. K. Hills, and to whose editorial section, we contributed occasionally, for fifteen years, begins Vol. 40 with H. Sheridan Baketel, A.M,, M.D, as editor We extend our best wishes and congratulations on the excellence of his first number to Dr. Baketel.

